# Supplementary figures and images for: Gene Ontology (GO)-Driven Inference of Candidate Proteomic Markers Associated with Muscle Atrophy Conditions
Source: Molecules. 2022 Aug 27;27(17):5514. doi: 10.3390/molecules27175514 (PMC9457532; doi:10.3390/molecules27175514)

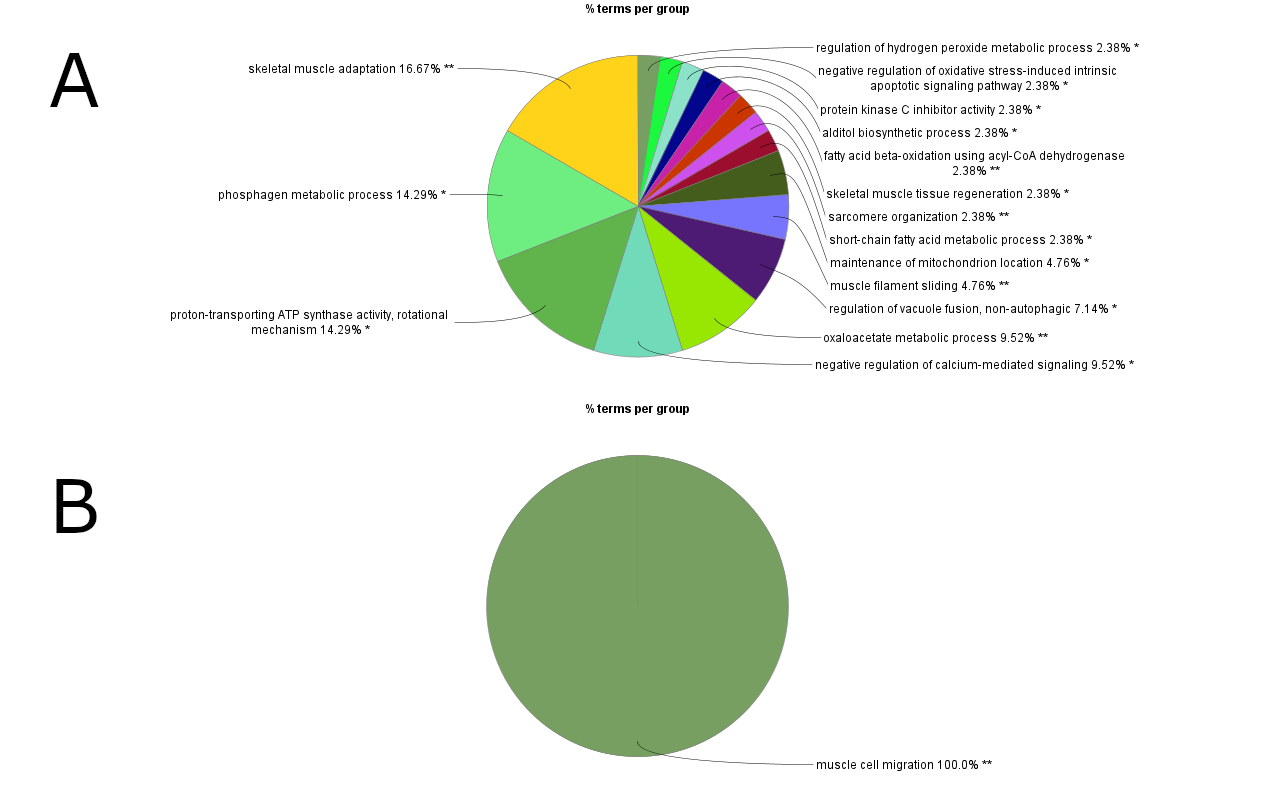

Supplement: Supplementary file 1 [file molecules-27-05514-s001.zip › Figure S1.jpg]
